# Cost analysis of treatment for periodontitis stages I–IV based on periodontal status and number of visits: a retrospective study at the Dental Teaching Hospital

**DOI:** 10.3389/fdmed.2025.1684749

**Published:** 2025-10-30

**Authors:** Aysha Azzahra B., Benso Sulijaya, Yuniarti Soeroso, Natalina Haerani, Fatimah Maria Tadjoedin, Anton Rahardjo

**Affiliations:** ^1^ Undergraduate Program, Faculty of Dentistry, Universitas Indonesia, Depok, Indonesia; ^2^ Department of Periodontology, Faculty of Dentistry, Universitas Indonesia, Jakarta, Indonesia; ^3^ Dental Division, Universitas Indonesia Hospital, Depok, Indonesia; ^4^ Department of Dental Public Health, Faculty of Dentistry, Universitas Indonesia, Jakarta, Indonesia

**Keywords:** treatment costs, periodontitis stages I–IV, periodontitis treatment, periodontal status, epidemiology

## Abstract

**Background:**

Periodontitis affects 20%–50% of the global population and 74.1% of the population in Indonesia, with 1.1 billion cases worldwide in 2019. Assessing treatment costs is essential to ensure affordability and optimize outcomes, particularly in developing countries.

**Objective:**

This study aimed to estimate the treatment cost for stage I–IV periodontitis, evaluate changes in periodontal parameters [plaque index (PI), papillary bleeding index (PBI), and calculus index (CI)], and analyze both the relationship and differences between treatment cost, number of visits, and changes in periodontal status.

**Materials and methods:**

A retrospective observational study of 64 patient records (2020–2022) from the Periodontology Clinic, Universitas Indonesia Dental Hospital, was conducted. The medical records of single-visit patients or patients who lacked data on the periodontal variables in their medical records were excluded. Data included treatment costs (during the initial, surgical, and supportive phases), number of visits, and periodontal parameters (PI, PBI, and CI). A univariate analysis was performed to describe the distribution of the treatment cost, number of visits, and periodontal parameters. A bivariate analysis was conducted using Pearson’s correlation to examine the association between treatment cost, number of visits, and changes in periodontal parameters, while one-way ANOVA was used to assess differences in the mean periodontal changes among the groups with different treatment costs and number of visits, with a significance threshold of *p* < 0.05. The statistical analyses were performed using SPSS 26.0.

**Results:**

The univariate analysis showed that the highest mean cost was incurred by those with stage IV periodontitis (USD 50.45), while the lowest was incurred by those with stage III periodontitis (USD 18.96). Regarding the treatment phases, the surgical phase incurred the highest mean cost (USD 61.21), whereas the initial phase incurred the lowest (USD 20.08). The bivariate analysis using Pearson’s correlation indicated no significant associations between treatment cost or number of visits and changes in PI, PBI, or CI (*p* > 0.05). Similarly, the one-way ANOVA revealed no significant differences in mean changes in PI, PBI, and CI across the groups based on treatment cost and number of visits (*p* > 0.05).

**Conclusion:**

Treatment cost and number of visits showed no significant association with periodontal status, suggesting that clinical outcomes are determined by treatment quality rather than expenditure or frequency of visits.

## Introduction

Periodontitis is a highly common disease in dental and oral patients. The prevalence of periodontal disease itself is 20%–50% globally (1). In 2019, there were 1.1 billion people worldwide affected by periodontitis (2). In Indonesia, based on data from the Basic Health Research survey in 2018, the prevalence reached 74.1% (3). Periodontitis is a prolonged inflammatory disease in the supporting tissues of the teeth that can lead to the destruction of gingival attachment, tooth loss, and abscesses (4, 5). Periodontitis is classified based on staging and grading (6). Staging classification focuses on describing the severity level or complexity of the periodontitis (6), while grading focuses on describing the progression of the disease (6). In this study, the analysis of treatment cost will focus on the staging classification. The staging classification itself consists of stages I, II, III, and IV (6). There are several stages in periodontitis treatment, namely initial evaluation, non-surgical or initial therapy, surgical therapy, and other treatment (6–9). In periodontitis cases, management is crucial to prevent the symptoms of the disease, such as cavities and tooth loss (10).

Issues in health programs and disease management have been persistent in Indonesia. Based on data from the Basic Health Research survey (Riskesdas) in 2018, 57.6% of the Indonesian population experienced oral health problems, with only 10.2% receiving medical treatment.

Among these, 14% were patients with periodontal disease, namely, gingivitis and periodontitis. One of the leading causes of this issue is the insufficient allocation of effectively placed funds by the government (11, 12). This was mainly due to a lack of knowledge of the cost of treatment that needs to be prepared and allocated in government health programs, which commonly occurs in developing countries (11, 12). Without proper knowledge of treatment cost, the government and other stakeholders will find it challenging to address these health issues, even if they have sufficient funds to finance the treatment of a particular disease (12). To generate cost estimation data, a cost of treatment analysis is conducted (13). This analysis involves estimating the cost of periodontitis treatment (cost) based on periodontal status (clinical outcomes) and the number of visits (13).

A treatment cost analysis is crucial for the health sector and the government (14). Both the health sector and the government can use the results of this analysis to formulate cost reduction strategies, estimate the economic burden of periodontitis management, and implement economic evaluations of specialized programs (15, 16). In 2019, the global economic burden from the cost of treatment and diseases related to periodontitis reached 52 million USD (17). In neighboring Malaysia, the disease burden amounted to a loss of 6.9 million USD (18). It is regrettable that in Indonesia, there is currently no data on the cost of periodontitis treatment. Therefore, this research was conducted to estimate the cost of periodontitis treatment based on periodontal status. The hope is that the findings of this study can serve as a justification for dental intervention programs, an economic framework for dental programs, and a representation of the funding allocation required by individuals, the government, agencies, and relevant stakeholders.

## Materials and methods

### Study design

This was a retrospective observational study that used medical records from the Periodontology Clinic, Dental Teaching Hospital, Faculty of Dentistry, Universitas Indonesia, from the past 2 years. This study was approved by the Commission of Ethical Research in Dentistry, Faculty of Dentistry, Universitas Indonesia, number: 33/Ethical Approval/Universitas Indonesia Faculty of Dentistry/VIII/2023, with protocol number: 010600823. The cost analysis of periodontitis treatment for stages I-IV in this study was based on changes in periodontal status, namely plaque index (PI), papillary bleeding index (PBI), and calculus index (CI), and the number of visits to the Dental Teaching Hospital, Faculty of Dentistry, Universitas Indonesia. Normality (Kolmogorov–Smirnov), correlation (Pearson), and group comparison tests (ANOVA) were included in the analysis, with significance thresholds of *p* < 0.05.

### Inclusion and exclusion criteria

This research focused on collecting data on patient outcomes after periodontal treatment to calculate the cost of periodontitis treatment. The criteria for inclusion were complete and legible medical records of patients who underwent all treatments from beginning to end from April 2020 to July 2022. The medical record should include the diagnosis of the patient’s periodontitis stage (I–IV) that required treatment at the Periodontology Clinic, Dental Teaching Hospital, Faculty of Dentistry, Universitas Indonesia.

Medical records with incomplete and illegible conditions were excluded, as were those of patients with a single visit, as subsequent visits were needed to analyze their treatment outcomes. Other exclusion criteria were medical records that lacked complete patient treatment data and those that lacked data on the variables (PI, PBI, and CI) from the patient's first visit and subsequent visits, as these variables were needed to analyze the changes in treatment outcomes from one visit to another.

### Periodontal status examination

All the parameters were obtained from the medical records. The PI used was from Greene and Vermillion (19). The PBI used was from Mühlleman (20). The CI used was from the Calculus Index-Simplified (CI-S) (21).

### Cost analysis

Treatment cost was defined as the overall treatment fee at the dental hospital and was analyzed to determine whether it had an effect on the treatment outcome or changes in PI, PBI, and CI (13). Indirect treatment costs, such as the patient's transportation fee or productivity costs, were not included. The treatment cost was listed as the billing cost in the medical records from April 2020 to July 2022. The treatment cost was originally in Indonesian rupiahs and was converted to dollars in December 2023 (1 USD = Rp 16,031.93) (22). The treatment sequence comprised a combination of the initial treatment, reconstructive treatment, surgical treatment, and other treatment phases that the patients underwent (23–26). The number of visits was defined as the total number of patient visits from the initial visit to the final visit that were recorded (14).

## Results

Medical records were successfully collected from the Periodontology Clinic of Dental Teaching Hospital, Faculty of Dentistry, Universitas Indonesia, for the period from April 2020 to July 2022. Out of 210 medical records, 64 medical records were eligible. Table 1 presents the data regarding the age, gender, and periodontitis stage of the patients. The groups with the largest number of patients were as follows: the 41–60 years of age category, female, periodontitis stage III, underwent the initial treatment sequence, and visited the clinic twice. Table 2 presents the percentage distribution of treatment sequences for each stage of periodontitis as recorded. For those with periodontitis stage I, the treatment sequences were evenly distributed. However, among those with stages II–IV, the initial treatment sequence was the most common. Table 3 shows the average total treatment cost based on the periodontitis stage and treatment phase. The highest average cost was found in stage IV, while the lowest cost was in stage III. The highest cost was incurred in the initial and surgery sequence, while the lowest cost was incurred in the initial treatment sequence. Table 4 presents the average changes in PI, PBI, and CI based on periodontitis stages and treatment sequences. The highest average change in PI and CI was found among those with periodontitis stage III, while PBI had the highest among those with stage I. The highest average changes in PI, PBI, and CI were found in the initial and other treatment, initial and surgery, and initial and reconstruction sequence groups, respectively. Table 5 presents an analysis of periodontitis treatment cost, which was calculated by dividing the average treatment cost by the average changes in PI, PBI, and CI. The lowest treatment costs in diagnosis and sequence are found in stage III and the initial sequence. In diagnosis, the lowest treatment cost analyses are found all in stage III and in the initial sequence.

**Table 1:** Characteristics of patients with stage I–IV periodontitis.

Variable	Category	Frequency (*n*)	Percentage (%)
Age	≤40 years	23	34.3
	41–60 years	26	38.8
	>60 years	18	26.9
Sex	Female	39	58.2
	Male	28	41.8
Periodontitis stage	Stage I	4	6.0
	Stage II	11	16.4
	Stage III	31	46.3
	Stage IV	21	31.3
Treatment sequence	Initial	50	0.8
	Initial and other treatment	7	0.1
	Initial and reconstruction	4	0.1
Number of visits	Initial and surgery	3	0.0
	2 times	29	45.3
	3 times	22	34.4
	4 times	4	6.3
	5 times	4	6.3
	6 times	2	3.1
	7 times	1	1.6
	8 times	1	1.6
	12 times	1	1.6

**Table 2:** Distribution of treatment sequences.

Treatment sequence	Periodontitis stage I (*n* = 4)	Periodontitis stage II (*n* = 10)	Periodontitis stage III (*n* = 31)	Periodontitis stage IV (*n* = 19)	Total (*n* = 64)
Initial	1 (1.56%)	8 (12.50%)	25 (39%)	16 (25%)	50 (78.13%)
Initial and other treatment	1 (1.56%)	2 (3.13%)	2 (3.13%)	2 (3.13%)	7 (10.94%)
Initial and reconstruction	1 (1.56%)	0 (0%)	2 (3.13%)	1 (1.56%)	4 (6.25%)
Initial and surgery	1 (1.56%)	0 (0%)	2 (3.13%)	0 (0.00%)	3 (4.69%)

**Table 3:** Distribution of treatment cost based on periodontitis stage and treatment sequence.

Variable	Treatment cost (USD)			
	Mean	Std. deviation	Minimum	Maximum
Periodontitis stage				
Stage I	41.78	23.62	15.88	73.07
Stage II	21.57	17.35	9.53	68.94
Stage III	18.96	16.79	6.35	82.60
Stage IV	50.45	49.14	9.53	162.02
Treatment sequence				
Initial	20.08	23.08	6.35	127.07
Initial and other treatment	46.74	27.86	15.88	88.95
Initial and reconstruction	59.01	59.42	22.87	147.72
Initial and surgery	61.21	11.91	49.24	73.07

**Table 4:** Distribution of changes in PI, PBI, and CI based on periodontitis stage and treatment sequence.

Variable	Mean Δ PI	Mean Δ PBI	Mean Δ CI
Periodontitis stage			
Stage I	0.575	1.343	0.729
Stage II	0.648	0.933	0.810
Stage III	0.862	0.930	0.820
Stage IV	0.791	1.045	0.704
Treatment sequence			
Initial	0.79	0.97	1.18
Initial and other treatment	1.04	1.21	1.10
Initial and reconstruction	0.80	1.15	1.38
Initial and surgery	0.95	1.48	1.33

**Table 5:** Analysis of periodontitis treatment costs from changes in PI, PBI, and CI based on periodontitis stage and treatment sequence.

Variable	Treatment cost (USD)/Mean *Δ* PI	Treatment cost (USD)/Mean *Δ* PBI	Treatment cost (USD)/Mean *Δ* CI
Periodontitis stage			
Stage I	72.65	31.12	57.27
Stage II	33.28	23.13	26.65
Stage III	21.99	20.39	23.12
Stage IV	63.79	48.29	71.70
Treatment sequence			
Initial	25.44	20.74	17.06
Initial and other treatment	45.07	38.68	42.39
Initial and reconstruction	73.99	51.42	42.76
Initial and surgery	64.20	41.36	45.91

Table 6 presents the results of Pearson’s correlation test, examining the correlation between treatment cost or number of visits and changes in PI, PBI, and CI. We found that there was no significant difference or relationship between treatment cost or number of visits and changes in PI, PBI, and CI, as the *p*-values were  > 0.05. In the ANOVA, all three variables had *p*-values  > 0.05. This indicates that there were no significant differences or relationships between treatment cost or number of visits and changes in periodontal status.

**Table 6:** Analysis of the relationship between treatment cost (USD) and number of visits and changes in PI, PBI, and CI in patients with periodontitis.

Periodontal status	Treatment cost (USD)		Number of visits	
	r	*p*	r	*p*
*Δ* PI	−0.084	0.512	0.106	0.402
*Δ* PBI	0.130	0.304	0.185	0.143
*Δ* CI	0.068	0.594	0.076	0.551

The Kolmogorov–Smirnov normality test (*n* > 50) showed a normal data distribution.

*Pearson’s correlation test; *p* < 0.05 indicates a significant value.

## Discussion

Among the 64 medical records, the age group with the highest prevalence of periodontitis (stages I–IV) was the 41–60 years age group. This result resembles that of a study conducted in China, in which the age group with the highest prevalence was 45–65 years (27). The number of female patients was higher than that of male patients in this study, constituting 58.2% of the total. Several studies have found that periodontitis generally occurs more frequently in men as they tend to have smoking habits and are more prone to diabetes (28–30). However, it is important to note that in this study, there were multiple inclusion criteria that needed to be met, and as a result, some patients with periodontitis were not included in this research.

The majority of the patients had stage III periodontitis, accounting for 46.3%. This result is consistent with a study that was conducted using medical records from the Dental Teaching Hospital, Faculty of Dentistry, Universitas Indonesia, from 2017 to 2019. That study also indicated that periodontitis was predominantly diagnosed at stage III, particularly in patients with smoking habits, hypertension, and diabetes (31). Table 1 indicates that the highest percentage for treatment sequence is the initial sequence, accounting 0.8% percentage of all other sequences. This is because initial treatment serves as the early phase of periodontal treatment and not all treatments require immediate surgical intervention to eliminate etiological factors, especially in the milder stages (31). The predominance of initial treatment reflects the conservative management of early-stage periodontitis and the fact that surgical interventions are reserved for complex cases (31). A majority of the patients visited the hospital twice. The number of visits varied considerably, indicating a diverse need for subsequent visits (32).

Stage IV periodontitis incurred the highest treatment cost, which aligned with various other studies (33). This phenomenon can be explained by the fact that stage IV periodontitis poses a higher risk to older patients and has a higher risk of tooth loss (33). The treatment and maintenance required to manage stage IV periodontitis are more extensive, hence the higher associated cost (33). The most expensive treatment sequence was the initial and surgical sequence, which is required to manage stage IV periodontitis (34). Table 4 presents the changes in PI, PBI, and CI among the patients. The highest change in these variables was found in those with stage III (for PI and CI) and stage IV (for PBI) periodontitis. This phenomenon can be explained by the effectiveness of surgical treatment in stage III and IV periodontitis compared to non-surgical treatment (35). This is due to the nature of surgical treatment, such as flap surgery, which has better access to deep periodontal pockets than non-surgical treatment, and thus has a better success rate in periodontal pocket reduction (35). The data are contrary to previous explanations, which stated that as the stage increases, the disease complications also increase (34). However, this could explain why treatment outcomes are better in stage III than in stage IV (34).

In a systematic review and meta-analysis, periodontal pockets with moderate (5–6 mm) or deep (>6 mm) depths are significantly reduced after patients undergo flap surgery compared to scaling and root debridement (35). This explains why the largest decrease in the variables occurred among those with stage III and IV periodontitis, which may be influenced by the age and sex of the patients (35). It is important to note that the data in this study were collected from patient visits over a full year (with potentially long intervals between visits), so the high effective clinical outcomes may not necessarily be directly attributed to surgical or other complex treatments. It was found in Table 5 that stage IV is the highest treatment cost for PBI and CI. This may be due to the cost of treatment and the effectiveness of complex treatments (36). However, it should be noted that highest cost for PI is held by stage I and the lowest costs for PBI and CI are held by stage III. This indicates that further research is still needed to clarify the explanations for this condition.

Tables 6, 7 present the Pearson’s correlation and ANOVA test results. The bivariate analysis examined the relationship between treatment cost or number of visits and changes in PI, PBI, and CI. The data obtained show that there was no significant relationship or difference between treatment cost or number of visits and changes in PI, PBI, and CI. This study concluded that treatment cost and number of visits do not always significantly influence treatment outcomes (PI, PBI, and CI). Thus, in resource-limited settings, it should be noted that these variables do not always contribute to the efficacy of treatment.

**Table 7:** Analysis of the relationship between treatment cost (USD) and number of visits and changes in PI, PBI, and CI in patients with periodontitis.

Periodontal status	Treatment cost (USD)	Number of visits
	*p*	*p*
*Δ* PI	0.988	0.514
*Δ* PBI	0.679	0.181
*Δ* CI	0.758	0.413

The Kolmogorov–Smirnov normality test (*n* > 50) showed a normal data distribution.

The homogeneity of variance test (*p* > 0.05) showed a homogeneous data distribution.

*ANOVA; *p* < 0.05 indicates a significant difference.

It should be noted that the cause of changes in PI, PBI, and CI was not further defined. For example, whether the changes were due to the diagnosis, treatment sequence, or even the treatment itself. In the future, further research should analyze more detailed variables, such as evaluating the outcomes of flap procedures with or without bone grafts (37). This could help researchers get more precise answers as to whether treatment cost, number of visits, and changes in PI, PBI, and CI are significantly related, especially if the analysis is conducted with a narrower scope.

This study has several limitations that need to be considered. First, the retrospective design relies on existing data, which may be incomplete and subject to bias. Additionally, the small effective sample size reduces the statistical power and limits the generalizability of the findings. Potential biases are also unavoidable, including selection bias and reliance on retrospectively collected data. Other than that, the study does not take into account the impact of a patient’s systemic health on periodontitis, which may lead to more treatment appointments and increase the cost of treatment. Finally, the lack of long-term follow-up restricts our ability to assess any outcomes that may appear over an extended period. Therefore, the results should be interpreted with these limitations in mind.

## Conclusion

The results of the analysis showed that the highest treatment cost, based on changes in PI, PBI, and CI, was incurred by those with stage I and stage IV periodontitis. The lowest treatment costs for all the treatment sequences were found among those with stage III periodontitis, based on changes in PI, PBI, and CI. The highest treatment cost, based on changes in PI and PBI, was for the initial and reconstruction sequence. When based on changes in CI, the highest treatment cost was for the initial and surgical sequence. The initial sequence incurred the lowest cost based on all the variables. This study found that there was no significant relationship between the cost of periodontitis treatment and changes in periodontal status (PI, PBI, and CI). Similarly, there was no significant relationship between changes in periodontal status (PI, PBI, and CI) and the number of visits.

## Data Availability

The raw data supporting the conclusions of this article will be made available by the authors, without undue reservation.

## References

[1] GBD 2017 Disease and Injury Incidence and Prevalence Collaborators. Global, regional, and national incidence, prevalence, and years lived with disability for 328 diseases and injuries for 195 countries, 1990–2016: a systematic analysis for the Global Burden of Disease Study 2016. Lancet. (2017) 390(10100):1211–59. 10.1016/S0140-6736(17)32154-2 28919117 PMC5605509

[2] Chen MX Zhong YJ Dong QQ Wong HM Wen YF. Global, regional, and national burden of severe periodontitis, 1990–2019: an analysis of the Global Burden of Disease Study 2019. J Clin Periodontol. (2021) 48(9):1165–88. 10.1111/jcpe.13506 34101223

[3] Apriani L Sunarjo L Widyawati M Wiguna R. Impact and non-pharmacological therapy of periodontitis in pregnant women; literature review. J Holist Nurs Health Sci. (2022) 5(1):125–44. 10.14710/hnhs.5.1.2022.125-144

[4] Nazir MA. Prevalence of periodontal disease, its association with systemic diseases and prevention. Int J Health Sci. (2017) 11(2):72–80. PMC5426403 28539867

[5] Ermawati T. Periodontitis and diabetes mellitus. Stomatognatic. J.K.G Unej. (2012) 9(3):152–4.

[6] Tonetti MS Greenwell H Kornman KS. Staging and grading of periodontitis: framework and proposal of a new classification and case definition. J Clin Periodontol. (2018) 45:S149–61. 10.1111/jcpe.12945 29926495

[7] Patel PK Jacobson R. Aesthetic Surgery of the Facial Skeleton. Philadelphia, PA: Elsevier (2022).

[8] Elashiry M Morandini AC Cornelius Timothius CJ Ghaly M Cutler CW. Selective antimicrobial therapies for periodontitis: win the “battle and the war”. Int J Mol Sci. (2021) 22(12):6459. 10.3390/ijms22126459 34208697 PMC8235535

[9] Peeran S Ramalingam K. Periodontal maintenance therapy. In: Newman MG Takei H Klokkevold PR Carranza FA, editors. Carranza’s Clinical Periodontology. 13th ed. St. Louis, MO: Elsevier (2021). p. 978–4–3.

[10] Mehrotra N Singh S. Periodontitis. In: StatPearls [Internet]. Treasure Island, FL: StatPearls Publishing (2023). Available online at: https://www.ncbi.nlm.nih.gov/books/NBK541126/ (Accessed May 1, 2023)

[11] Curtis B Evans RW Sbaraini A Schwarz E. Geographic location and indirect costs as a barrier to dental treatment: a patient perspective. Aust Dent J. (2007) 52:271–5. 10.1111/j.1834-7819.2007.tb00501.x 18265681

[12] Akin J Birdsall N de Ferranti D. Financing Health Services in Developing Countries. Washington, DC: The World Bank (1987).

[13] dos Santos Silva EK Cruz JAW da Cunha MAVC Azevedo B dos Santos LMP dos Santos Neto PM Cost-effectiveness in health: consolidated research and contemporary challenges. Humanit Soc Sci Commun. (2021) 8:254. 10.1057/s41599-021-00940-5

[14] Mohd-Dom T Ayob R Mohd-Nur A Abdul-Manaf MR Ishak N AbdulMuttalib K Cost analysis of periodontitis management in public sector specialist dental clinics. BMC Oral Health. (2014) 14:56. 10.1186/1472-6831-14-56 24884465 PMC4033493

[15] Probandari A. Cost effectiveness analysis in health policy determination: just a concept or applicable? J Manaj Pelayanan Kesehat. (2011) 14(01):47–51. Available online at: https://journal.ugm.ac.id/index.php/jmpk/article/viewFile/2712/2435

[16] Grosse SD. Assessing cost-effectiveness in healthcare: history of the $50,000 per QALY threshold. Expert Rev Pharmacoecon Outcomes Res. (2008) 8(2):165–78. 10.1586/14737167.8.2.165 20528406

[17] Petersen PE Bourgeois D Ogawa H Estupinan-Day S Ndiaye C. The global burden of oral diseases and risks to oral health. Bull World Health Org. (2005) 83(9):661–9. 16211157 PMC2626328

[18] Dom TNM Ayob R Abd Muttalib K Aljunid SM. National economic burden associated with management of periodontitis in Malaysia. Int J Dent. (2016) 2016:1891074. 10.1155/2016/1891074 27092180 PMC4820592

[19] Greene JC Vermillion JR. Oral hygiene index: a method for classifying oral hygiene status. J Am Dent Assoc. (1960) 61(5):172–9. 10.14219/jada.archive.1960.0177

[20] Mühlemann HR. Psychological and chemical mediators of gingival health. J Prev Dent. (1977) 4:6–17. 275483

[21] Reddy S. Essentials of Clinical Periodontology and Periodontics. 3rd ed. New Delhi: Jaypee Brothers Medical Publishers (2011).

[22] ExchangeRates UK. US Dollar to Indonesian Rupiah History: 2024. London: ExchangeRates UK (2025). Available online at: https://www.exchangerates.org.uk/USD-IDR-spot-exchange-rates-history-2024.html (Accessed September 21, 2025)

[23] Ajay M Negi KS Saroj T Kanwarjeet AS. A successfully treated case of severe periodontitis using interdisciplinary approach: report of a case. J Indian Soc Periodontol. (2016) 20(1):95–7. 10.4103/0972-124X.168496 27041848 PMC4795146

[24] Silva JC Muniz FWMG Oballe HJR Andrada ACF Rösing CK Cavagni J The effect of non-surgical periodontal therapy on oxidative stress biomarkers: a systematic review and meta-analysis. J Clin Periodontol. (2018) 45(2):139–53. 10.1111/jcpe.12993 30076616

[25] Gasner NS Schure RS. Periodontal disease. In: StatPearls [Internet]. Treasure Island, FL: StatPearls Publishing (2023). Available online at: https://www.ncbi.nlm.nih.gov/books/NBK554590/ (Accessed April 10, 2023)

[26] Cepeda MS Weinstein R Blacketer C Lynch MC. Association of flossing/inter-dental cleaning and periodontitis in adults. J Clin Periodontol. (2017) 44(9):866–71. 10.1111/jcpe.12765 28644512 PMC5601277

[27] Huang Q Dong X. Prevalence of periodontal disease in middle-aged and elderly patients and its influencing factors. Am J Transl Res. (2022) 14(8):5677–84. 36105065 PMC9452307

[28] Ioannidou E. The sex and gender intersection in chronic periodontitis. Front Public Health. (2017) 5:189. 10.3389/fpubh.2017.00189 28824898 PMC5543279

[29] Eke PI Dye BA Wei L Thornton-Evans GO Genco RJ. Prevalence of periodontitis in adults in the United States: 2009 and 2010. J Dent Res. (2012) 91(10):914–20. 10.1177/0022034512457373 22935673

[30] Shiau HJ Reynolds MA. Sex differences in destructive periodontal disease: a systematic review. J Periodontol. (2010) 81(10):1379–89. 10.1902/jop.2010.100044 20450376

[31] Tadjoedin ESS Dewi NP Soeroso Y Sulijaya B Natalina N. Stage and grade determination of periodontitis accompanied by systemic conditions and diseases according to American Academy of Periodontology 2017 classification: study at Dental Hospital, Faculty of Dentistry, Universitas Indonesia. J Dentomaxillofac Sci. (2021) 6(2):88–93. 10.15562/jdmfs.v6i2.1205

[32] Pasupuleti MK Subhadra Penmetsa G Gangolu M Ramesh Konathala SV Naga Venkata Satya S. Role of communication, professionalism, and clinical care skills of postgraduate students on patients recall visits in dental school an observational study. J Patient Exp. (2020) 7(6):1563–7. 10.1177/2374373520942984 33457615 PMC7786739

[33] Schwendicke F Krois J Engel AS Seidel M Graetz C. Long-term periodontitis treatment costs according to the 2018 classification of periodontal diseases. J Dent. (2020) 99:103417. 10.1016/j.jdent.2020.103417 32592828

[34] Chatzopoulos G Wolff LF Lunos S Patwari P Sumanth S Garcia M Association between periodontitis extent, severity, and progression rate with systemic diseases and smoking: a retrospective study. J Pers Med. (2023) 13(5):814. 10.3390/jpm13050814 37240984 PMC10223170

[35] Salehuddin NQ Sabri BAM Ariffin F. Patients’ view on non-surgical and surgical periodontal therapy in relation to oral health: a narrative review. Dentistry Review. (2022) 2(3):100058. 10.1016/j.dentre.2022.100058

[36] Serino G Rosling B Ramberg P Socransky SS Lindhe J. Initial outcome and longterm effect of surgical and non-surgical treatment of advanced periodontal disease. J Clin Periodontol. (2001) 28(10):910–6. 10.1034/j.1600-051x.2001.028010910.x 11686808

[37] Vernazza C Heasman P Gaunt F Pennington M. How to measure the costeffectiveness of periodontal treatments. Periodontol 2000. (2012) 60:138–46. 10.1111/j.1600-0757.2011.00406.x 22909111

